# Dieckol from *Ecklonia cava* Regulates Invasion of Human Fibrosarcoma Cells and Modulates MMP-2 and MMP-9 Expression via NF-*κ*B Pathway

**DOI:** 10.1155/2011/140462

**Published:** 2011-08-04

**Authors:** Chen Zhang, Yong Li, Zhong-Ji Qian, Sang-Hoon Lee, Yong-Xin Li, Se-kwon Kim

**Affiliations:** ^1^Department of Chemistry, Pukyong National University, Busan 608-737, Republic of Korea; ^2^Key Laboratory of Molecular Enzymology and Enzyme Engineering of Ministry Education, College of Life Science, Jilin University, Changchun 130021, China; ^3^Marine Bioprocess Research Center, Pukyong National University, Busan 608-737, Republic of Korea; ^4^School of Pharmaceutical Sciences, Changchun University of Chinese Medicine, Changchun 130117, China

## Abstract

The matrix metalloproteinase (MMP) family is involved in the breakdown of extracellular matrix in normal physiological processes, as well as in the disease processes such as arthritis and cancer metastasis. In the present study, dieckol was obtained with high yield from marine brown alga *Ecklonia cava* (EC), and its effect was assessed on the expression of MMP-2 and -9 and morphological changes in human fibrosarcoma cell line (HT1080). Dieckol inhibited the expression of MMP-2 and -9 in a dose-dependent manner and also suppressed the cell invasion and the cytomorphology in 3D culture system on HT1080 cells. Moreover, dieckol may influence nuclear factor kappa B (NF-*κ*B) pathway without obvious influence on activator protein-1 (AP-1) pathway and tissue inhibitor of metalloproteinases (TIMPs). In conclusion, dieckol could significantly suppress MMP-2 and -9 expression and alter cytomorphology of HT1080 cell line via NF-*κ*B pathway.

## 1. Introduction

The past few decades of cancer research has revealed that matrix metalloproteinases (MMPs) play a significant role in a variety of pathologic conditions. Especially in malignant tumors, the activities of MMPs are deregulated and their expressions are often associated with poor prognosis. Among all the MMPs, MMP-2 and -9 have demonstrated to play a major role in the establishment of metastasis, which substantially increases in majority of malignant tumors. Therefore, inhibition of MMP-2 and -9 is thought to have a therapeutic benefit for cancer [1–8]. 

**Figure 1:**
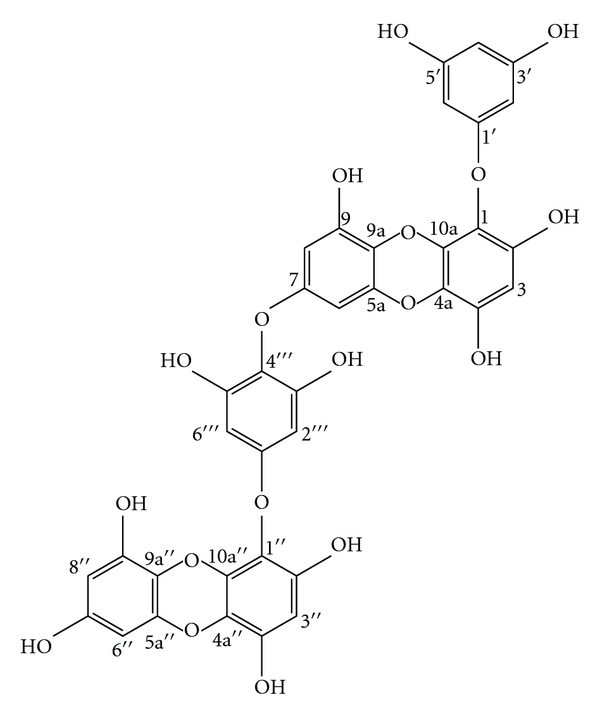
Chemical structure of dieckol.

Recently, significant achievements have been made in synthetic bioactive applications. However, natural compounds and their derivatives are still known as the richest source of bioactive compounds with huge pharmaceutical application potential [9]. Dieckol (Figure 1) is a phloroglucinal derivative isolated from marine brown alga *Ecklonia cava *(EC) with a variety of biological functions *in vitro* and *in vivo*, for example, antioxidant, antitumor, antihuman immunodeficiency virus (-HIV), and anti-inflammatory activities. For instance, dieckol is a novel safe phloroglucinal derivative which inhibited the cytopathic effects of HIV-1 including HIV-1-induced syncytia formation, lytic effects, and viral p24 antigen production, as well as exhibited reverse transcriptase enzymes inhibitory and HIV-1 entry activity in addition to its other biological properties [10, 11].

In the present study, effect of dieckol on the expression of MMP-2 and -9, cytotoxicity, and cellular invasiveness was evaluated in HT1080 cells. The selectivity of this cell line was based on the extensive studies performed previously on MMPs expression [12]. In addition, the mechanism of dieckol regulating MMP-2 and -9 via the influence on nuclear factor kappa B (NF-*κ*B) pathway has also been investigated. 

## 2. Materials and Methods

### 2.1. General Materials

^1^H NMR (400 MHz) and ^13^C NMR (100 MHz) spectra were recorded on a JEOL JNM-ECP 400 NMR spectrometer (JEOL, Japan), using DMSO-*d*_6_ solvent peak (2.50 ppm in ^1^H and 39.5 ppm in ^13^C NMR) as an internal reference standard. For some signals, the chemical shifts approximated at the third decimal place. This is to distinguish between signals of very close value, but which could nevertheless be clearly differentiated by visual inspection of the spectra. MS spectra were obtained on a JEOL JMS-700 spectrometer (JEOL, Japan). Extraction of EC was performed using extraction unit (Dongwon Scientific Co., Republic of Korea). Column chromatography was carried out by silica gel 60 (230–400 mesh, Merck, Germany) and Sephadex LH-20 (Sigma, St. Louis, Mo). Thin-layer chromatography (TLC) was run on precoated Merck Kieselgel 60 F_254_ plates (0.25 mm), and the spots on the TLC plate were detected under UV lamp (254 and 365 nm) using CHCl_3_/MeOH/H_2_O/acetic acid (65 : 25 : 4 : 3, v/v/v/v) as a development solvent system. Vanillin-H_2_SO_4_ was employed as the detecting agent for phenolic compounds. All the solvent and chemicals used in this study were of reagent grade from commercial sources (Duksan Pure Chemicals Co., Ltd., Republic of Korea). 

### 2.2. Extraction, Isolation, Purification, and Elucidation of Dieckol

*Ecklonia cava* was collected from Jeju Island coast of Republic of Korea, washed three times with water to remove salt, and subjected to lyophilization. The lyophilized EC was ground into powder before extraction. The dried EC powder (10 kg) was extracted by stirring extraction unit with MeOH (3 × 5 L) for 10 days at room temperature (25°C). The extract (273 g) was suspended in water and partitioned with n-hexane (35.92 g), CH_2_Cl_2_ (20.49 g), EtOAc (24.87 g), and n-BuOH (106 g) in a sequence. The EtOAc fraction (24.87 g) was subjected to a silica gel flash chromatography eluted with Hexane/EtOAc/MeOH/CH_2_Cl_2_ (gradient) to yield ten subfractions (F1–F10). The F5 subfraction was further purified by Sephadex LH-20 with MeOH obtained dieckol (58.30 mg).

### 2.3. Cell Culture and Cell Viability Assay

Human fibrosarcoma cells (HT1080) from ATCC were cultured in Dulbecco's Modified Eagle's Medium (DMEM), supplemented with 10% fetal bovine serum (FBS), 2 mM glutamine, and 100 U/mL penicillin-streptomycin, and incubated at 5% CO_2_ and 37°C humidified atmosphere. For experiments, cells were passaged at least for 5 times and detached with Trypsin-EDTA. For cell viability assay, cells were seeded in 96-well plates at a density of 1 × 10^4^ cells/well in 200 *μ*L DMEM containing 10% fetal bovine serum. After 24 hours, the medium was removed and the cells were incubated for 48 h with DMEM containing 1% FBS in the absence or presence of various concentrations of dieckol. Forty-eight hours later, 100 *μ*L 3-(4,5)-dimethylthiahiazol (-z-y1)-3,5-di-phenytetrazoliumromide (MTT) (0.5 mg/mL final concentration) was added to each well and incubated for another 4 hours at 37°C in 5% CO_2_. Finally, DMSO (150 *μ*L) was added to solubilize the formazan salt formed and amount of formazan salt was determined by measuring the OD at 540 nm using GENios microplate reader (Tecan Austria GmbH, Austria). Relative cell viability was determined by the amount of MTT converted into formazan salt. Viability of cells was quantified as a percentage compared to the control and dose response curves were developed. The data were expressed as mean (±SD) from three independent experiments.

### 2.4. *In Vitro* Invasion Assay

An *in vitro* invasion assay was performed using a 24-well transwell unit (8 *μ*m pore size) with polyvinylpyrrolidone-free polycarbonate filters coated with 500 *μ*g/mL of Matrigel and placed in transwell chambers. The coated filters were washed thoroughly in PBS and dried immediately before use. Cells were placed in the upper part of the transwell plate and allowed cell attachement for 24 hours, then incubated with dieckol for 36 hours at 37°C. The cells that invaded the lower surface of the membrane were fixed with methanol and stained with 0.5% crystal violet for 10 min. Finally, we determined invasive phenotypes by counting the cells that migrated to the lower side of the filter using Leica DM6000B microscopy at 200 × (Leica Microsystems Wetzlar GmbH, Germany) in at least 5 bright fields [13].

### 2.5. Three-Dimensional (3D) Culture of HT1080 Cell Line

The cells behavior and morphology in 3D culture system is quite different from that observed in the 2D system [14]. The 3D culture model was established as described previously [15]. Briefly, HT1080 cells (1.5 × 10^3^) were suspended with a neutralized solution of type I collagen (1 mg/mL) (Sigma, St. Louis, Mo) and 1/5 volume of a 5 × DMEM. The cell suspension containing various final concentrations of dieckol was added to 24-well plates and kept at 37°C until gelled. The plates were then incubated at 37°C for 24 hours. The results were observed under microscope at 200 × (Leica Microsystems Wetzlar GmbH, Germany).

### 2.6. Gelatin Zymography

For gelatin zymography, HT1080 cells were seeded in 24-well plates using serum-free media and pretreated with different concentrations of dieckol for 1 hour. MMP expression was stimulated by 12-O-tetradecanoylphorbol 13-acetate (PMA) (10 ng/mL) and incubation was continued for 48 hours. After incubation, conditioned media were collected and their protein contents were determined by the Bradford method [16]. After normalizing the protein content, equal amounts of proteins were electrophoresed under nonreducing conditions on 10% polyacrylamide gels containing 1.5 mg/mL gelatin. Following electrophoresis, polyacrylamide gels were washed with 50 mM Tris-HCl (pH 7.5) containing 2.5% Triton X-100 to remove sodium dodecyl sulfate. Gels were then incubated overnight at 37°C in a developing buffer containing 10 mM CaCl_2_, 50 mM Tris-HCl, and 150 mM NaCl to digest gelatin by MMPs. Areas of gelatin hydrolyzed by MMPs were visualized as clear zones against blue background by Coomassie blue staining and the intensities of the bands were estimated by densitometry (Multi Gauge V3.0 software, Fujifilm Life Science, Japan) [12].

### 2.7. Reverse Transcription-PCR

Total RNA from each sample was isolated by TRIzol reagent and subjected for reverse transcription-PCR (RT-PCR). For RT-PCR analysis, total RNA was reverse-transcribed to synthesize cDNA using a commercial kit (TaKaRa RNA PCR kit (AMV)) according to the manufacturer's protocol. PCR was then carried out in 50 *μ*L of reaction volumes containing RNA PCR buffer, 2.5 mM MgCl_2_, 0.2 *μ*M of each primer, and 2.5 units of TaKaRa Taq polymerase. Samples were predenatured at 94°C for 4 min, followed by amplification at 94°C for 1 min, at 55°C for 30 s, and at 72°C for 1 min for 25 cycles, followed by a final 10 min extension step at 72°C. The primers for GAPDH were 5′-GTCAACGGATTTGGTCGTATT-3′′ and 5′-AGTCTTCTGGGTGGCAGTGAT-3′ with an expected amplified product of 300 bp. The MMP-2 primers were 5′-CTCAGATCCGTGGTGAGATCT-3′ and 5′-CT  TTGGTTCTCCAGCTTCAGG-3 with an expected amplified product of 496 bp. The MMP-9 primers were 5′-ATCCAGTTTGGTGTCGCGGAGC-3′ and 5′-GAAGGGGAAGACGCACA  GCT-3′ with an expected amplified product of 552 bp.

### 2.8. Extraction of Nuclear and Plasma Proteins and Western Blot Analysis

HT1080 cells were pretreated without or with different concentrations of dieckol for 1 hour followed by the stimulation with PMA, and the incubation was continued for another 48 hours. For separate extraction of nuclear and cytoplasm proteins, CelLytic NuCLEAR Extraction kit (S26-36-23, Sigma-Aldrich Co., Mo, USA) was used by following manufacturer's instructions. The cells were lysed with 0.5 mL of lysis buffer (500 *μ*L hypotonic lysis buffer, 5 *μ*L 0.1 M dithiothreitol (DTT), and 5 *μ*L protease inhibitor cocktail, proteasome inhibitor, MG132) for 15 min on the ice. Igepal CA-630 solution (4 *μ*L) was added and vortexed for 20 s. nuclei were separated by centrifugation at 10,000 × g for 10 min and supernatant (cytoplasm protein) was collected. Precipitated nuclei were lysed with 100 *μ*L of extraction buffer mix (98 *μ*L of extraction buffer, 1 *μ*L of 0.1 M, DTT and 1 *μ*L of protease inhibitor cocktail) for 10 min and nuclei protein were collected by centrifugation at 12,000 × g for 10 min. Proteins were separated by 12% SDS-PAGE and electrotransferred onto PVDF membranes (Millipore, Bedford, Mass). After blocking with 5% skim milk in PBST (PBS, pH 7.6, containing 0.2% Tween-20), the membranes incubated with primary antibody (Santa Cruz Biotechnology, Inc. us). for 1 hours, washed with 0.2% Tween-20 in PBS, and then incubated with horseradish peroxidase-linked secondary antibody (Santa Cruz Biotechnology, Inc., Calif, USA). The reactive signals were visualized by ECL kit (PE Applied Biosystems).

### 2.9. Statistical Analysis

All the experiments were repeated at least three times. All results were expressed as the mean of three replicates determination and standard deviation (SD). Statistical comparisons were made with Student's *t*-test and *P* values <0.05 were considered to be significant.

## 3. Results

### 3.1. Cell Viability Assay

**Figure 2:**
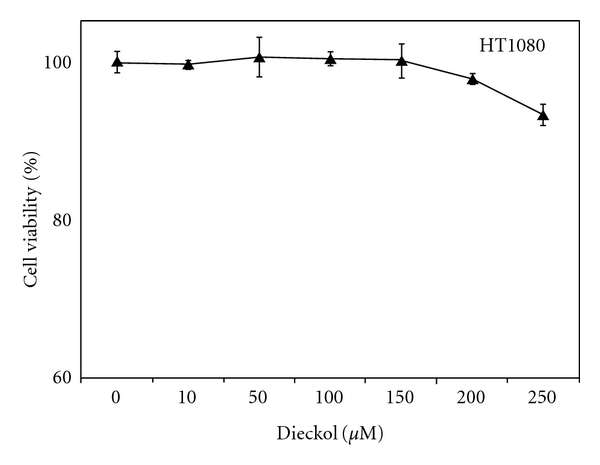
Effect of dieckol on the viability of HT1080 cells. Data represented as mean ± SD of three independent experiments.

Cell viability assay was introduced for determining whether dieckol can have toxic effect on HT1080 cells at high concentration. Comparison of cell growth over 48 h with various dieckol concentrations (0–250 *μ*M) were showed in Figure 2. The values for all concentrations were similar with control (dieckol 0 *μ*M) indicating that dieckol does not affect cell viability below the concentrations of 200 *μ*M.

### 3.2. Transwell Invasion Assay

**Figure 3:**
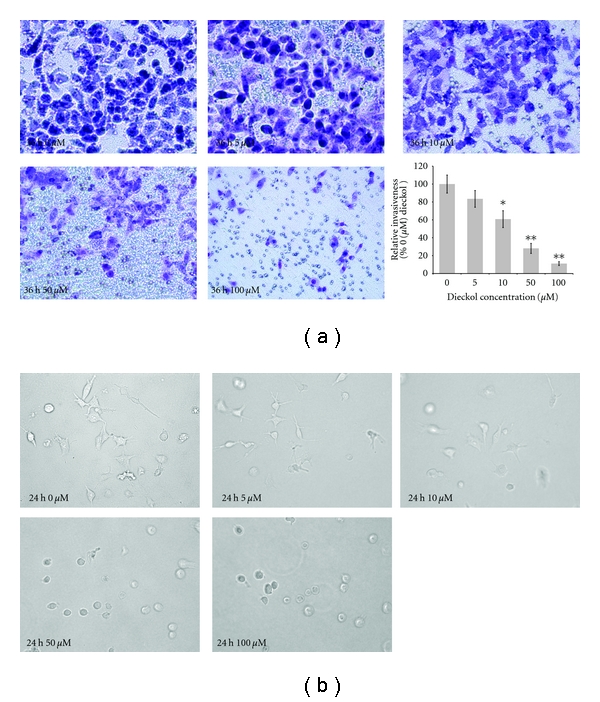
Effect of dieckol on invasion in HT1080 cells. (a) Microphotographs of filters of the Matrigel chamber invasion assay. Inset (a) represents the conclusive data of cellular invasion. (b) Dieckol Effect on the morphology of HT1080 cells in three-dimensional (3D) cultures system. The data presented are mean ± SD of three independent experiments. Values are compared with the 0 *μ*M dieckol, which are significant at *P < 0.05 and **P < 0.01.

Although no cytotoxic effect was observed even below 200 *μ*M concentration of dieckol on HT1080 cell line, ideally, 0 to 100 *μ*M of dieckol was used in the cell invasion and the subsequent experiments. To investigate whether dieckol inhibits tumor invasion, Matrigel invasion assays were performed for dieckol-treated HT1080 cells. It was observed that dieckol treatment reduced the cell invasion, and 100 *μ*M of dieckol concentration significantly blocked tumor invasion (Figure 3(a)) [13, 17].

### 3.3. Effects of Dieckol on the 3D Culture in HT1080 Cell Line

To investigate the effect of dieckol on the 3D culture system of HT1080 cells, same density of cells were seeded into 3D collagen gel with various final concentrations of the dieckol. After 36 h, the control (without dieckol) stretched out in the gel and formed many branched and elongated structures in type I collagen matrix (Figure 3(b)). In contrast, the branches were reduced in the cells treated with 5 *μ*M concentration of dieckol. As the concentration increased to 100 *μ*M, these branches disappeared and cells changed to spherical shape.

### 3.4. Effects of Dieckol on the MMP-2, -9 Transcription and Expression Levels in HT1080 Cell Line

**Figure 4:**
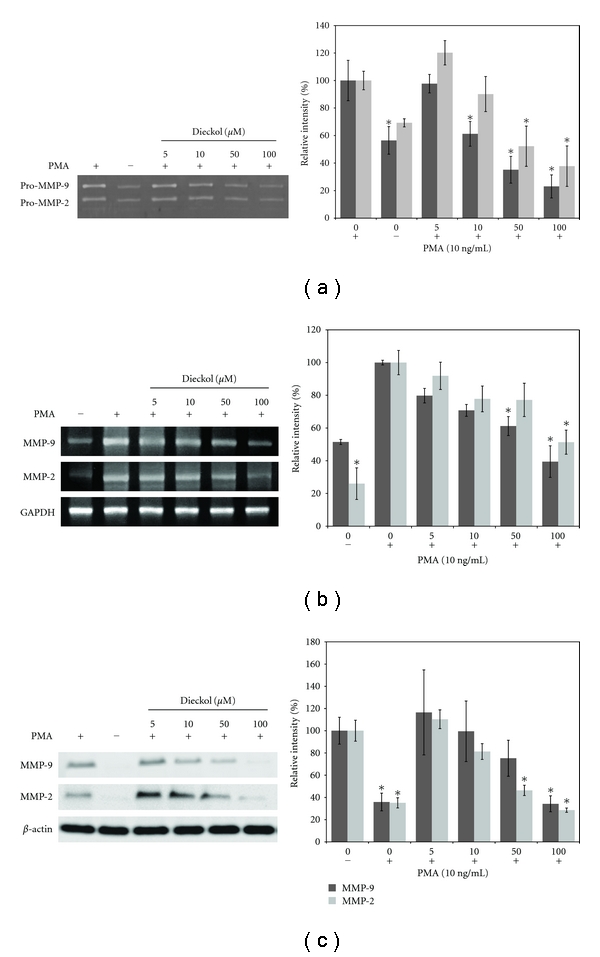
Effects of dieckol on the MMP expression. (a) Expression of MMP-2, -9 in dieckol-treated HT1080 cells by gelatin zymography. (b) mRNA transcription levels of MMP-2, -9 in HT1080 cells by RT-PCR analysis. (c) MMP-2, -9 protein expressions in the dieckol-treated HT1080 cells by western blot analysis. The columns presented in (a), (b), and (c) were the representative of three independent experiments. *, *P *< 0.05, Compared with the 0 *μ*M dieckol with PMA 10 ng/mL.

Gelatin zymography analysis was performed, for investigating whether dieckol could inhibit protein levels of MMP-2 and MMP-9. As shown in Figure 4(a), protein expressions for MMP-2 and MMP-9 were significantly inhibited by dieckol in a dose-dependent manner. In addition, results from the RT-PCR and western blot revealed that the expression of MMP-2 and -9 was inhibited at transcription levels by dieckol, more or less similar manner with zymography analysis (Figures 4(b) and 4(c)).

### 3.5. Effects of Dieckol Influenced AP-1, NF-*κ*B, and I*κ*B Expressions on HT1080 Cell Line

**Figure 5:**
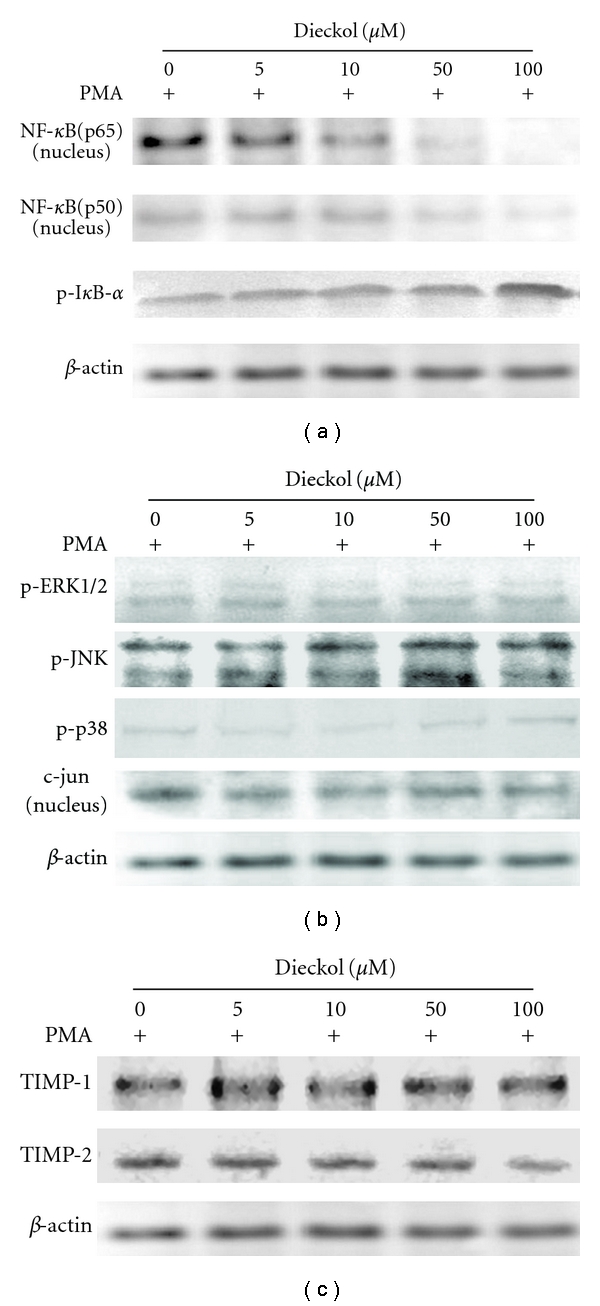
Effect of dieckol on the NF-*κ*B and AP-1 cell signaling pathway and TIMP-1, -2 expressions in HT1080 cells. (a) Nucleus NF-*κ*B (p65 and p50) and I*κ*B (p-I*κ*B-*α*) protein expressions in the dieckol treated HT1080 cells. (b) (c-jun), MAPK pathway (ERK, JNK, p38). (c) Western blot analysis of TIMP-1 and TIMP-2.

Western blot studies were carried out to assay the downregulation effects of dieckol on the possible mechanism of expression of activator protein-1 (AP-1) and nuclear factor-kappa B (NF-*κ*B). Meanwhile, the tissue inhibitor of metalloproteinases (TIMP) was been checked by western blot. As shown in Figure 5(b), dieckol did not exhibit a clear influence of activator protein-1 (AP-1) (c-jun) expression and mitogen-activated protein kinase (MAPK) (ERK, JNK, p38), which mediated the AP-1. The NF-*κ*B transcription family proteins consist of several protein subunits, and it was clear that the level of NF-*κ*B (p65 and p50) was decreased in a dose-dependent manner. The p-I*κ*B-*α* expression was increased significantly, when treated with high concentration of dieckol (100 *μ*M) (Figure 5(a)). As observed in Figure 5(c), there is no distinct change in the expressions of TIMP-1 and TIMP-2 when treated with different concentrations of dieckol.

## 4. Discussion

MMPs, a family of zinc-dependent endopeptidases, participate in many physiological and pathological processes. An imbalance between the inhibition and activation of MMPs is related to some diseases such as osteoarthritis, rheumatoid arthritis, tumor metastasis, cardiovascular diseases and congestive heart failure [18–20]. Finding MMP inhibitors (MMPIs) for treatment of these prevalent diseases has been an important aspect of present day cancer research [21]. MMPIs regulate MMPs at several biochemical pathways, but most of them directly inhibit the activity of enzyme. Till now, MMPIs entering clinical trial are only of synthetic origin (organic compounds) and have failed to enter the pharmaceutical market owing to their side effects [22]. The search for novel and natural compounds has led to the discovery of oceans as the treasure house of substances with amazing pharmacological potential. Therefore, discovering the ideal MMPIs from marine natural sources is feasible and more advantageous [23].

*Ecklonia cava* (a marine alga), one of the marine floral members, produces dieckol which has been well studied previously to inhibit the expression of various MMPs [12, 24]. In this study, dieckol did not show any cytotoxic effect on HT1080 cells at the concentrations below 200 *μ*M. However, dieckol influenced the cell mobility as measured by transwell invasion assay; it also suppressed HT1080 cells crossing the matrix gel in a dose-dependent manner. In addition, we have examined the effect of dieckol on the organization of HT1080 cells in a 3D culture environment. *In vivo*, cells experience a 3D environment and are surrounded by other cells and ECM. The traditional 2D monolayer culture system cannot emulate the complexities of the 3D tissue microenvironment and will always represent a suboptimal milieu for studying physiological ECM interactions with connective tissue cells [25, 26]. MMPs also affect cell morphology in 3D culture environment. MMPs cleave the matrix proteins fibronectin, vitronectin, and collagen I into smaller fragments. The cleaved ECM fragments then facilitate and accelerate cancer cell adhesion and invasion [15]. HT1080 cells formed the stellate structures in the 3D culture environment, and these structures migrated into the collagen matrix cleaving the ECM with the help of the MMPs. However, dieckol treatment significantly reduced the number and length of stellate structures. The cultured HT1080 cells were treated with dieckol with selected concentrations (0–200 *μ*M) and the expression of MMP-2, -9 was induced by PMA. Several studies have shown that PMA can induce expression of MMP-2, -9 [27]. Dieckol inhibited the expression of MMP-2, -9 in a dose-dependent manner, according to the results of RT-PCR, western blot and zymography assays. 

AP-1 and NF-*κ*B are major transcription factors that regulate MMP-2, -9 expression [28–30]. Members of the MAPK superfamily (ERK1/2, JNKs, and p38 kinase) are known to regulate AP-1 transactivation by increasing the level of AP-1 components or altering the phosphorylation of their subunits (c-jun, c-fos) and then regulate MMPs expression and activity in various cell types [31]. NF-*κ*B, in its inactive form, it is sequestered in the cytoplasm, bound by members of the inhibitor of kappa B (I*κ*B) family of inhibitor proteins (including I*κ*Ba, I*κ*Bb, I*κ*Bg, and I*κ*Be). Various stimuli that activate NF-*κ*B result in phosphorylation of I*κ*B, is followed by its ubiquitination and subsequent degradation [32, 33]. Dieckol suppress NF-*κ*B (p65 and p50) expression in a dose-dependent manner. However, dieckol did not show any clear effect on AP-1 and ERK1/2, JNKs, and p38 kinase expressions. It has been found that p-I*κ*B-*α* expression increased when treated with high concentration of dieckol (100 *μ*M), but not in low concentration [34]. It may suggest that the inhibitor of kappa B family could be upregulated by high concentration of dieckol, and this may be another reason for NF-*κ*B suppression by dieckol. Western blot resulted from TIMP-1 and TIMP-2 depicted that TIMP have not been influenced by different concentrations of dieckol. It is indicating that inhibition of MMP-2, -9 activity by dieckol is not because of the TIMP at the test concentrations.

At the promoter site of MMP-2 and -9 genes, several putative binding sites for transcription factors are present, which regulate gene expression. This promoter contains an AP-1 binding consensus site at −79 upstream from the starter site and further upstream, there is a cluster of regulatory elements including another AP-1 binding site called AP-2 and NF-*κ*B binding site [35]. It is commonly known that MMP-9 transcription is mainly regulated via AP-1 [36, 37]. In addition to this, under some inflammatory and other pathology conditions, MMP-2, -9 transcription can be regulated via NF-*κ*B signaling pathways. Interestingly, dieckol inhibited NF-*κ*B expression with a similar dose-dependent manner as observed for MMP-2, -9 expressions. However, it did not significantly influence the expression of AP-1. This mode of action may be contributed to the unique chemical structure of dieckol [38–40]. The reasons could be discussed based on the structural features of the isolated dieckol and the structure-activity relationship could be described due to its unique skeleton. For example, the number of hydroxyl groups present in its chemical structure probably plays an important role because of the higher polymerization of phloroglucinal units and on the other hand, the O-bridge linkages (ether linkage) among phloroglucinals donate more free anions to attract responsible receptors.

## 5. Conclusion

Dieckol was isolated from an edible marine brown alga *Ecklonia cava *has been characterized according to the MS and NMR data. It acts as an inhibitor of MMP-2, -9 expressions by the downregulation of NF-*κ*B pathway without significant influence on AP-1, MAPK pathway. Furthermore, the modulation of MMP-2 and -9 expressions could be one reason for the suppression of invasiveness and 3D culturing of HT1080 cell line. Hence, this study suggested that dieckol could be an effective candidate for the suppression of cancer invasion.
